# Mouse fat storage‐inducing transmembrane protein 2 (FIT2) promotes lipid droplet accumulation in plants

**DOI:** 10.1111/pbi.12678

**Published:** 2017-01-18

**Authors:** Yingqi Cai, Elizabeth McClinchie, Ann Price, Thuy N. Nguyen, Satinder K. Gidda, Samantha C. Watt, Olga Yurchenko, Sunjung Park, Drew Sturtevant, Robert T. Mullen, John M. Dyer, Kent D. Chapman

**Affiliations:** ^1^ Center for Plant Lipid ResearchUniversity of North TexasDentonTXUSA; ^2^ Department of Molecular and Cellular BiologyUniversity of GuelphGuelphONCanada; ^3^ US Arid‐Land Agricultural Research CenterUSDA‐ARSMaricopaAZUSA; ^4^ Present address: Department of Molecular GeneticsUniversity of TorontoTorontoONCanada; ^5^ Present address: Biology DepartmentCentral Arizona CollegeMaricopaAZ85138USA

**Keywords:** endoplasmic reticulum, fat storage‐inducing transmembrane protein 2, lipid droplets, lipid storage, triacylglycerol, lipid partitioning

## Abstract

Fat storage‐inducing transmembrane protein 2 (FIT2) is an endoplasmic reticulum (ER)‐localized protein that plays an important role in lipid droplet (LD) formation in animal cells. However, no obvious homologue of FIT2 is found in plants. Here, we tested the function of FIT2 in plant cells by ectopically expressing mouse (*Mus musculus*) FIT2 in *Nicotiana tabacum* suspension‐cultured cells, *Nicotiana benthamiana* leaves and *Arabidopsis thaliana* plants. Confocal microscopy indicated that the expression of FIT2 dramatically increased the number and size of LDs in leaves of *N. benthamiana* and *Arabidopsis,* and lipidomics analysis and mass spectrometry imaging confirmed the accumulation of neutral lipids in leaves. FIT2 also increased seed oil content by ~13% in some stable, overexpressing lines of *Arabidopsis*. When expressed transiently in leaves of *N. benthamiana* or suspension cells of *N. tabacum*, FIT2 localized specifically to the ER and was often concentrated at certain regions of the ER that resembled ER‐LD junction sites. FIT2 also colocalized at the ER with other proteins known to be involved in triacylglycerol biosynthesis or LD formation in plants, but not with ER resident proteins involved in electron transfer or ER‐vesicle exit sites. Collectively, these results demonstrate that mouse FIT2 promotes LD accumulation in plants, a surprising functional conservation in the context of a plant cell given the apparent lack of FIT2 homologues in higher plants. These results suggest also that FIT2 expression represents an effective synthetic biology strategy for elaborating neutral lipid compartments in plant tissues for potential biofuel or bioproduct purposes.

## Introduction

Lipid droplets (LDs) are organelles found in all types of plant cells where they function to compartmentalize neutral lipids such as triacylglycerols (TAGs), steryl esters (SEs) and even isoprenoids (e.g. carotenoids, rubber) (Chapman and Ohlrogge, 2012; Murphy, 2001). Once considered as simply a repository for lipid storage molecules, these organelles are becoming more appreciated for their roles in other dynamic cellular processes, including signalling, trafficking and membrane remodelling (Brasaemle and Wolins, 2012; Chapman *et al*., 2012). LDs have a ‘half‐unit’ membrane of phospholipids covering a hydrophobic core of nonpolar lipids, and in different cell types, different proteins localize to the LD surface (Murphy, 2012). Perhaps best studied in plants are the oleosins associated with LDs in seed tissues (Chapman *et al*., 2012; Frandsen *et al*., 2001; Huang, 1996), but a number of other proteins are located on the surface of LDs as well, including the recently identified lipid droplet‐associated proteins (LDAPs) that appear to stabilize LDs in many nonseed (i.e. vegetative) cell types (Chapman *et al*., 2012; Gidda *et al*., 2016; Horn *et al*., 2013; Kim *et al*., 2016).

Cytosolic LDs, in both seeds and nonseed tissues, have also been referred to in the literature as lipid bodies, oil bodies, oleosomes and spherosomes, but more recently, the term lipid droplet has been used to emphasize a more cohesive and evolutionary relationship with neutral‐lipid‐containing organelles from various cell types and organisms (Murphy, 2012). Cytosolic LDs in plant cells are distinctly different in location and composition from plastoglobuli, which are LDs that originate from the thylakoid membrane and accumulate in the plastid stroma (Austin *et al*., 2006; Nacir and Bréhélin, 2013). By contrast, cytosolic LDs are believed to originate from the endoplasmic reticulum (ER), where the neutral lipids are synthesized and partitioned somehow between the membrane bilayer leaflets before being packaged for release from the ER surface (Wanner *et al*., 1981). In plants, storage lipid accumulation into LDs has mostly been studied in seed tissues (Chapman *et al*., 2012), and transcriptional regulation of lipid synthesis and compartmentation is modulated through several key transcription factors, including *WRINKLED1* (*WRI1*) and *LEAFY COTYLEDON2* (*LEC2*) (Sreenivasulu and Wobus, 2013). Although a number of specific proteins in yeast, invertebrates and mammals have been identified that participate in LD ontogeny (Beller *et al*., 2010; Gao and Goodman, 2015; Pol *et al*., 2014), relatively little is known mechanistically about how plant cells form LDs at the ER and facilitate their release into the cytosol in seeds or nonseed tissues.

There are several models for cytosolic LD biogenesis in eukaryotic cells, and these all involve the ER in some manner. A widely accepted ‘lens’ model indicates that TAGs collect within the ER membrane bilayer and then bud from the ER surface into the cytosol to form an individual LD (Choudhary *et al*., 2015). In another model, pre‐existing vesicles are filled with neutral lipids from the ER to form LDs (Walther and Farese, 2009). Yet another model suggests that LDs that are formed from ER‐derived TAGs remain attached to the ER, perhaps even being interconnected with the ER lumen, and enlarging or reducing in size and prevalence based on cellular needs (Farese and Walther, 2009; Jacquier *et al*., 2011; Mishra *et al*., 2016). Others have suggested the possibility that LDs grow by small LDs fusing together to form large LDs (Wilfling *et al*., 2014). And finally, an ‘egg‐in‐cup’ model has been proposed where LDs originate at a particular location on the cytosolic surface of the ER membrane, whereby the ER partially surrounds the developing LD in such a way that it resembles an LD‐’egg’ inside an ER‐membrane ‘cup’ (Robenek *et al*., 2006). Although these various models represent different mechanistic views of how the LD organelle is produced, one shared feature is that LDs are formed in association with the ER. Indeed, the ways in which LDs form in cells likely include several mechanisms that are cell type‐ or stage‐specific and may not be mutually exclusive (Chapman *et al*., 2012; Pol *et al*., 2014; Wilfling *et al*., 2014).

The ER is the subcellular location for the majority of glycerolipid assembly within cells and includes enzymes for the synthesis of the neutral lipid LD core, as well as the phospholipid monolayer of the LD surface (Chapman and Ohlrogge, 2012; Chapman *et al*., 2012). In addition, a number of membrane‐bound proteins, mostly identified in animals and yeast, have been shown to be involved in the biogenesis of LDs and localized at the ER, often at ER‐LD junctions, or even on nascent LDs (Brasaemle and Wolins, 2012; Pol *et al*., 2014; Walther and Farese, 2009; Wang *et al*., 2016; Wilfling *et al*., 2014). One of the proteins that has been implicated in the sequestration of TAG for LD formation is the fat storage‐inducing transmembrane protein (FIT) (Kadereit *et al*., 2008). There are two isoforms of the FIT protein in mammals, namely FIT1 and FIT2. FIT1 is found in skeletal muscle and is less evolutionarily conserved than FIT2. FIT2 homologues, on the other hand, are found in organisms ranging from *Saccharomyces cerevisiae* to humans and in mammals are expressed predominantly in adipose tissue (Gross *et al*., 2010; Miranda *et al*., 2014). Ectopic overexpression of FIT2 in mammals induced the accumulation of large‐sized LDs at higher levels than are normally found and in tissues that are not normally LD‐rich (Gross *et al*., 2010; Kadereit *et al*., 2008). FIT2 overexpression did not, however, increase the synthesis of TAGs directly, but rather promoted the more efficient partitioning of neutral lipids from the ER membrane into nascent LDs (Gross *et al*., 2011; Kadereit *et al*., 2008). Notably, modifications of the FIT2 polypeptide sequence that reduced or enhanced TAG‐binding affinity resulted in the production of less or more LDs, respectively (Gross *et al*., 2011). Further, knock‐down of FIT2 expression in adipose cells, zebrafish and mice reduced the accumulation of LDs (Goh *et al*., 2015; Gross *et al*., 2011; Kadereit *et al*., 2008; Miranda *et al*., 2014).

FIT2 was shown recently to be essential for the budding of nascent LDs from the ER into the cytosol in yeast and mammalian cells (Choudhary *et al*., 2015), and disruption of *FIT2* in *Caenorhabditis elegans* was lethal (Choudhary *et al*., 2015). Interestingly, no homologues of FIT2 have been detected in the genomes of plants (Chapman *et al*., 2012). The importance of FIT2 in promoting LD formation in other eukaryotes prompted us to ask whether mammalian FIT2 would/could function to promote LD biogenesis in a plant cell context. Towards that end, we expressed the FIT2 protein from mouse (*Mus musculus*) in two transient plant cell systems, *Nicotiana tabacum* suspension‐cultured cells and *Nicotiana benthamiana* leaves, as well as via stable expression in transgenic *Arabidopsis thaliana* plants. Overall, our results indicate that FIT2, as it does in other eukaryotes, localizes to the ER in plant cells, including regions of the ER that appear to be involved in TAG biosynthesis and LD formation. FIT2 also promotes the accumulation of LDs in plant cells that normally do not accumulate substantial amounts of LDs, like those in leaves. We conclude that FIT2 likely interacts with the native LD biosynthetic machinery in plant cells to promote enhanced neutral lipid compartmentalization and the budding of LDs from ER, and its ectopic expression represents a novel strategy to elaborate the neutral lipid compartment in plants for various biotechnology‐oriented applications.

## Results

### Mouse FIT2 induces the accumulation of cytosolic LDs in transiently transformed tobacco leaves

**Figure 1:**
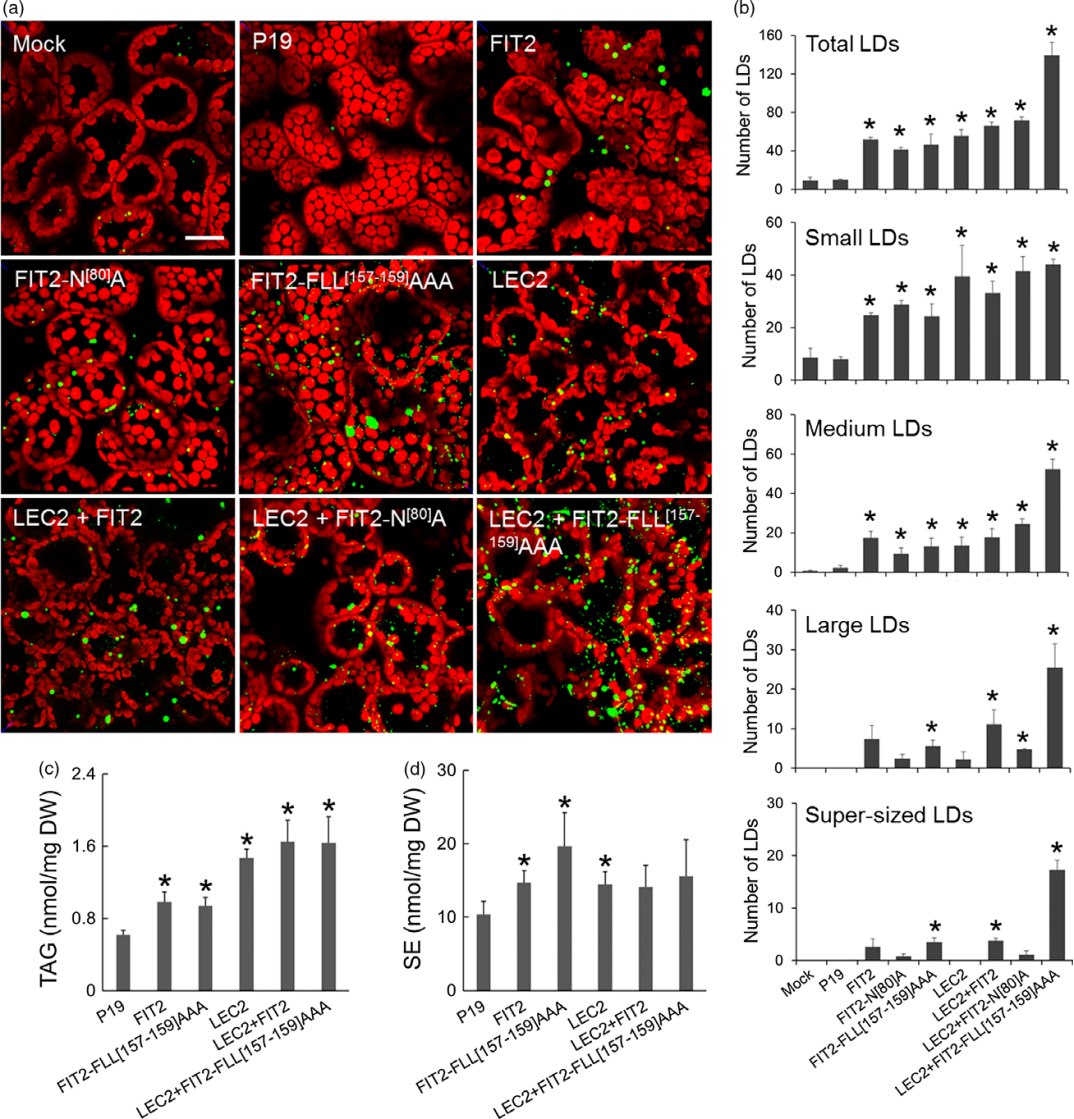
FIT2 expression in *Nicotiana benthamiana* leaves elaborates a neutral lipid compartment. (a) Representative confocal images of *N. benthamiana* leaves expressing FIT2 or mutated FIT2 proteins (i.e. FIT2‐N^[80]^A or FIT2‐FLL ^[157‐159]^ AAA) in the presence or absence of LEC2. Cells were (co)transformed as indicated by the panel label, and LDs (green) were visualized by staining with BODIPY 493/503. Red colour corresponds to chloroplast autofluorescence. Images are 2D projections of Z‐stacks of 50 optical sections. Bar = 20 μm. (b) Number of total LDs and LDs in different size categories per image area. The numbers and sizes of LDs were quantified by ImageJ as BODIPY‐stained lipid area in 2D projections of Z‐stacks (see Experimental procedures for details). Small LDs: BODIPY‐stained lipid area <3 μm^2^. Medium LDs: 3–6 μm^2^. Large LDs: 6–10 μm^2^. Super‐sized LDs: >10 μm^2^. Averages and SDs were calculated from three biological replicates (three Z‐stacks from each replicate). Asterisks indicate significant difference relative to the mock control at *P *≤* *0.05 determined by Student's *t*‐test. (c) and (d) TAG and SE content in infiltrated *N. benthamiana* leaves. Averages and SDs were plotted from four biological replicates. Asterisks indicate significant difference in comparison with the P19 control at *P *≤* *0.05 determined by Student's *t*‐test.

The FIT2 coding sequence from mouse was expressed in *Agrobacterium*‐infiltrated tobacco leaves to evaluate the effect of FIT2 expression on LD proliferation in a plant cell context (Figure 1). As shown in Figure 1a, confocal images of BODIPY 493/503‐stained LDs in mock (infiltration buffer only)‐transformed leaves or leaves that were expressing only the P19 viral protein (serving as an enhancer of transgene expression and included in all infiltrations [Petrie *et al*., 2010]) showed few LDs distributed throughout the cytosol (Figure 1a), which is typical of mesophyll cells in *N. benthamiana* leaves (Cai *et al*., 2015; Gidda *et al*., 2016). Similarly, chlorophyll autofluorescence in mock‐ and P19‐transformed cells revealed the expected distribution of chloroplasts, which mostly surrounded the cell's large central vacuole (Figure 1a). By contrast, expression of FIT2 in leaf cells resulted in a marked increase in both the numbers and sizes of LDs (Figure 1a, quantified in Figure 1b), all of which appeared to be localized within the cytosol and not in chloroplasts, indicating they were distinct from plastoglobuli. Further, expression of a FIT2 variant (FIT2‐N^[80]^A) known to have reduced TAG‐binding affinity compared to native FIT2 (Gross *et al*., 2011) resulted in an increase in the number of LDs in comparison with mock or P19 controls, but relatively fewer medium (3–6 μm^2^), large (6–10 μm^2^) or so‐called super‐sized (>10 μm^2^) LDs in comparison with cells expressing the native FIT2 protein. Expression of a FIT2 variant with enhanced TAG‐binding activity (i.e. FIT2‐FLL^[157‐159]^AAA; Gross *et al*., 2011), on the other hand, resulted in a further increase in super‐sized LDs in comparison with native FIT2 (Figure 1a and b).

As shown also in Figure 1a, ectopic expression of the LEC2 transcription factor, which induces genes for seed oil like synthesis in leaves (Santos Mendoza *et al*., 2005), resulted in an increase in LD proliferation, as expected (Cai *et al*., 2015; Gidda *et al*., 2016). While the total numbers of LDs induced by LEC2 expression were similar to that of FIT2, the numbers of large and super‐sized LDs were conspicuously greater with FIT2 in comparison with LEC2 (Figure 1b). These data suggest that FIT2 promotes the formation of larger‐sized LDs in plant cells. Indeed, combined expression of LEC2 and FIT2, particularly with the ‘enhanced’ FIT2 variant (FLL^[157‐159]^AAA), exaggerated further the total numbers of LDs in leaves, with a greater proportion of large and super‐sized LDs, as well as medium‐sized LDs, than with either FIT2 alone or LEC2 alone (Figure 1a and b). Moreover, FIT2 cooperated with the LEC2‐induced machinery to package ~2.5 times more neutral lipids (i.e. TAGs and some SEs) in these leaves (Figure 1c and d). Detailed analysis of the TAG and SE molecular species by conventional, direct‐infusion ESI‐MS/MS (electrospray ionization tandem mass spectrometry) (Figure S1) indicated that, generally, the TAGs and SEs that are normally present in leaves were all increased upon FIT2 expression. These same molecular species of TAG and SE were exaggerated further by co‐expression of FIT2 and LEC2, making the overall increase in TAGs and SE even more obvious (Figure S1). Notably, there was no further increase in TAG and SE in leaves co‐expressing LEC2 and the enhanced FIT2 variant (FLL^[157‐159]^AAA) in comparison with amounts observed in leaves co‐expressing LEC2 and native FIT2 (Figure 1c), despite a nearly twofold difference in the total number of LDs (Figure 1b). These observations would be consistent, however, with a primary role for FIT2 in the binding and partitioning of TAG into LDs, rather than a role in stimulating TAG synthesis (Gross *et al*., 2011; Kadereit *et al*., 2008).

### FIT2 localizes to the ER in plant cells and is enriched in ER subdomains involved in TAG assembly and LD biogenesis

**Figure 2:**
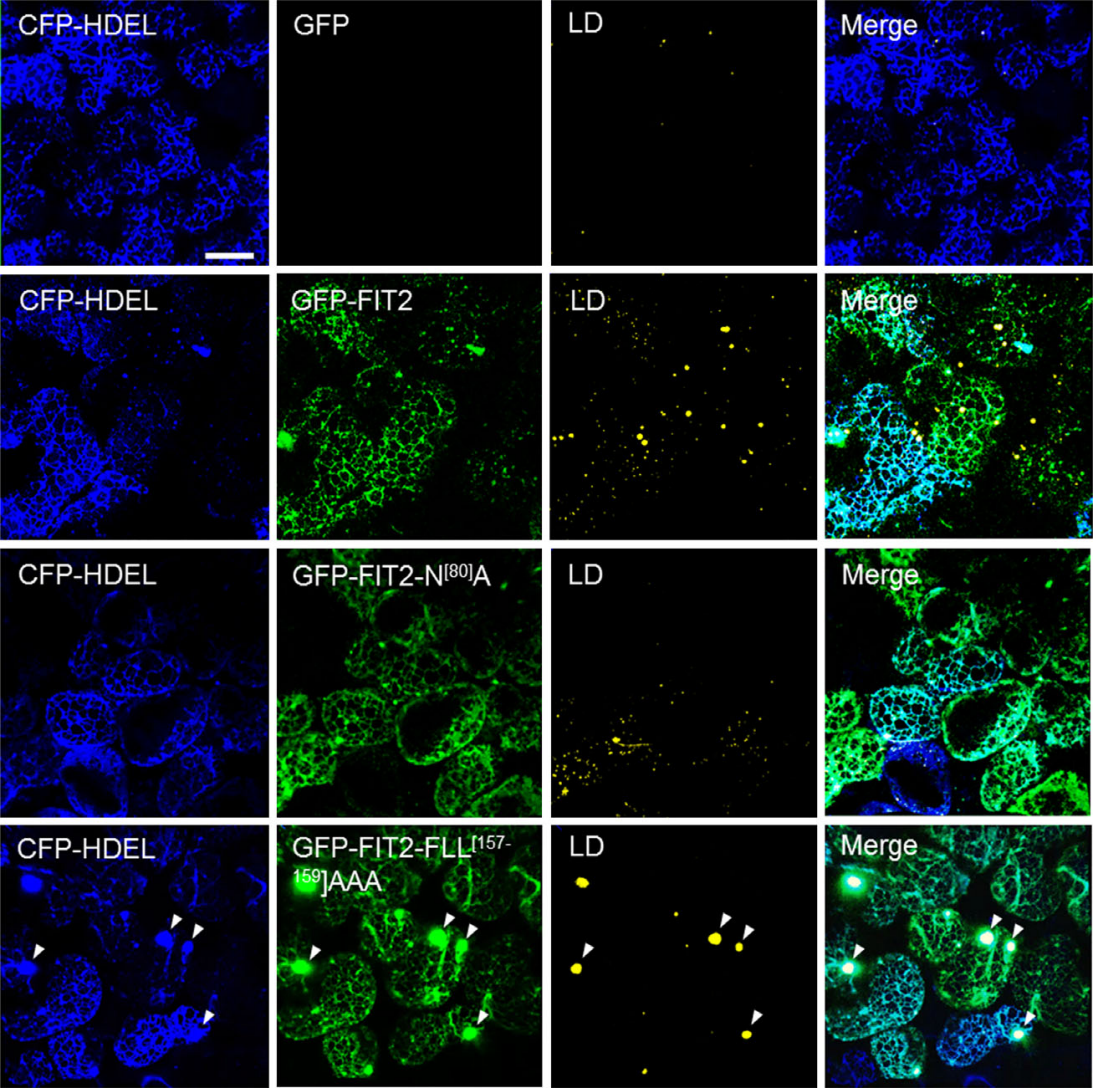
FIT2 and mutated FIT2 GFP‐tagged fusion proteins localize to the ER in *Nicotiana benthamiana* leaf mesophyll cells. As indicated by panel labels, ER, GFP‐FIT2/FIT2 variants and LDs were visualized (via confocal microscopy) in mesophyll cells based on fluorescence signals of CFP‐HDEL (blue), N‐terminal‐GFP‐tagged FIT2 or FIT2 variants (i.e. FIT2‐N^[80]^A or FIT2‐FLL ^[157‐159]^ AAA) (green) and Nile red (yellow), respectively). Shown also are the corresponding merged images. Note that no GFP fluorescence signal was detectable in the cell expressing CFP‐HDEL alone (top row), as expected. Arrowheads in the bottom row indicate examples of regions of the ER with altered morphology, where both GFP‐FIT2‐FLL ^[157‐159]^ AAA and Nile red‐stained LDs appear to accumulate. All images are 2D projections of Z‐stacks of ~30 slices. Bar = 20 μm.

The proliferation of LDs in plant cells following FIT2 expression suggested that it was functioning at the subcellular level in a manner similar to that in animal and yeast cells. Indeed, as shown in Figure 2, confocal imaging revealed that when GFP‐tagged FIT2 was expressed in *N. benthamiana* leaves, it localized exclusively to the ER, as evidenced by colocalization with a co‐expressed ER marker protein, CFP‐HDEL (Szymanski *et al*., 2007). Notably, GFP‐FIT2 and CFP‐HDEL co‐expressing cells also displayed a marked increase in the abundance of Nile red‐stained LDs when compared to cells expressing CFP‐HDEL alone (Figure 2), consistent with the induction of LDs in tobacco leaves transiently expressing FIT2 (Figure 1a and b). GFP‐tagged versions of FIT2‐N^[80]^A or FIT2‐FLL^[157‐159]^AAA also localized to the ER in tobacco leaf cells (Figure 2), and the elaboration of LDs in these cells was distinct from each other and that of wild‐type GFP‐FIT2, similar to results obtained using their non‐GFP‐tagged counterparts (Figure 1). That is, leaf cells expressing GFP‐FIT2‐N^[80]^A or GFP‐FIT2‐FLL^[157‐159]^AAA, relative to those expressing GFP‐FIT2, appeared to possess fewer or more larger‐sized LDs, respectively (Figure 2). Cells expressing GFP‐FIT2‐FLL^[157‐159]^AAA also displayed several conspicuous alterations in ER morphology, whereby the fusion protein (and CFP‐HDEL) localized not only throughout the normal, reticular‐like ER network, but also accumulated in specific regions of the ER that appeared globular in shape and were not observed in cells expressing CFP‐HDEL alone (Figure 2; refer to arrowheads in bottom row). These ER structures typically colocalized with or next to Nile red‐stained LDs, suggesting that these might be ER‐LD junction sites that formed as a result of excessive TAG binding and/or inefficient release of the nascent LDs from the ER, or simply might be a reflection of a normal process that was exaggerated by ectopic expression. It is possible also that the LDs with TAG may be trapped in the ER lumen at these locations (Mishra *et al*., 2016) or wrapped in ER membranes (Choudhary *et al*., 2015), but the resolution of light microscopy prevents more conclusive statements to be made about the nature of these structures.

**Figure 3:**
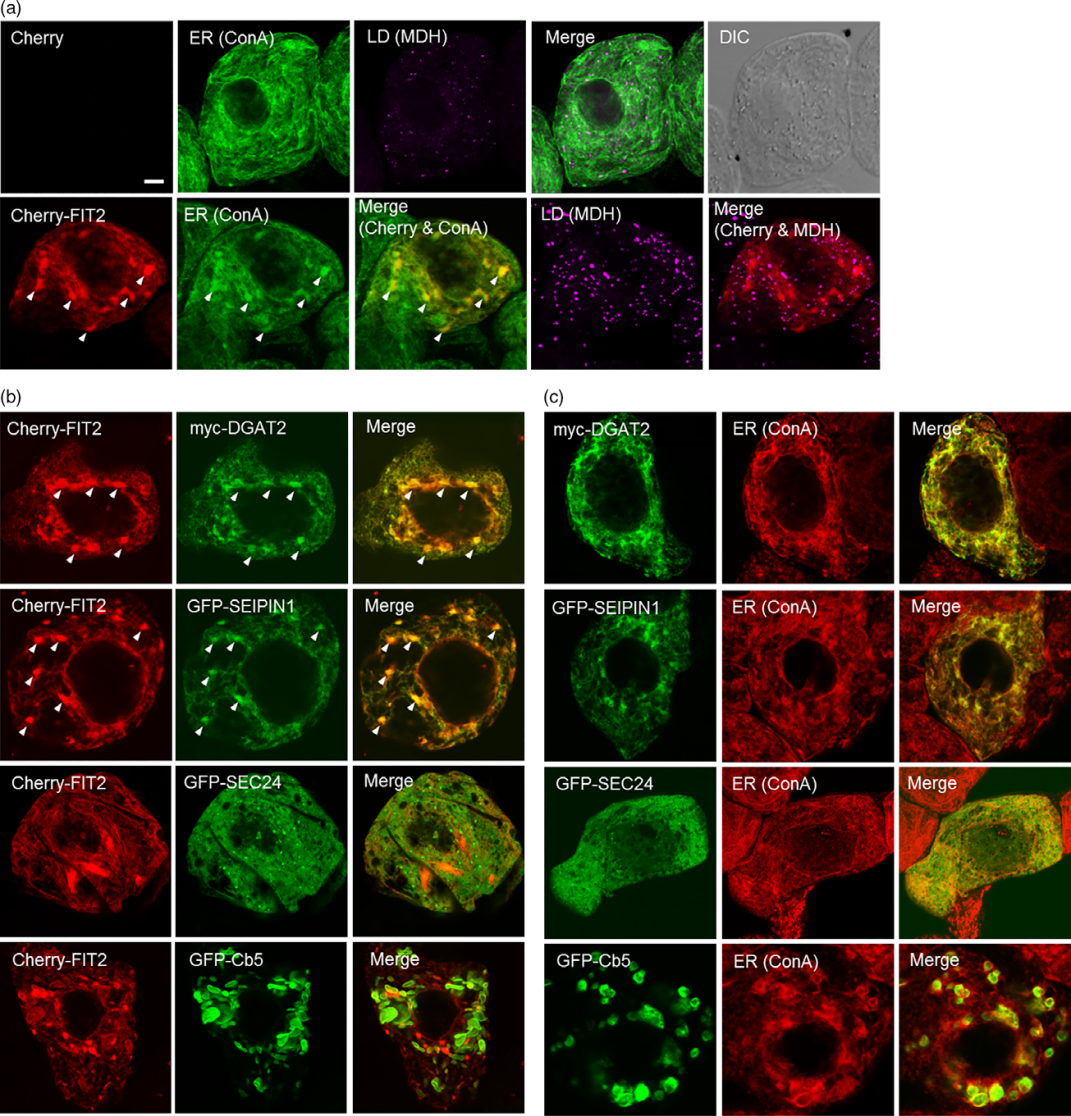
FIT2 expressed in *Nicotiana tabacum* suspension‐cultured BY‐2 cells is enriched in ER domains that also accumulate proteins involved in TAG biosynthesis and LD biogenesis. BY‐2 cells were transiently (co)transformed with the indicated proteins, and then, cells were visualized using confocal microscopy. (a) Representative confocal images showing the ER and LDs in mock‐transformed BY‐2 cells (top row), whereby the ER was stained using fluor‐conjugated ConA and LDs were stained using MDH (false‐coloured magenta). Shown also is the corresponding merged and differential interference contrast (DIC) images. The bottom row shows the fluorescence‐staining pattern of transiently expressed Cherry‐FIT2 in comparison with ConA‐stained ER and MDH‐stained LDs. Arrowheads in the bottom row indicate examples of Cherry‐FIT2 enriched in specific regions of ConA‐stained ER. Note that no Cherry fluorescence signal was detected in the mock‐transformed cell (top row), as expected. Bar = 5 μm. (b) Confocal images showing the localizations of Cherry‐FIT2 and various other co‐expressed ER‐localized proteins, including myc‐tagged DGAT2 or GFP‐tagged SEIPIN1, SEC24 or Cb5. Shown also are the corresponding merged images for each pair of co‐expressed proteins. Arrowheads illustrate examples of colocalization of FIT2 with DGAT2 and SEIPIN1. (c) Confocal images showing the localization of individually expressed Myc‐DGAT2 or GFP‐tagged SEIPIN1, SEC24 or Cb5 at the ConA‐stained ER. Shown also are the corresponding merged images for each protein and ConA‐stained ER.

We tested next whether mouse FIT2 could colocalize with other proteins known to be involved in TAG synthesis and/or LD formation in plant cells. For this purpose, we employed tobacco (*N. tabacum*) Bright Yellow 2 (BY‐2) suspension‐cultured cells, which serve as a well‐established model system for protein localization studies (Brandizzi *et al*., 2003; Miao and Jiang, 2007) and, given their relatively large size, are amenable to microscopic analysis of ER subdomains (Gidda *et al*., 2011; Hanton *et al*., 2007; Shockey *et al*., 2006). The proteins tested for colocalization included *Vernicia fordii* (tung tree) diacylglycerol acyltransferase 2 (DGAT2), which is known to be involved in TAG synthesis and localized to ER subdomains (Shockey *et al*., 2006), and *Arabidopsis* SEIPIN1, which localizes to ER‐LD sites where it is thought to promote the compartmentalization of neutral lipids into nascent, large‐sized LDs from the ER (Cai *et al*., 2015). As shown in Figures 3a and S2, and similar to the results presented above for GFP‐FIT2 in *N. benthamiana* leaf cells (Figure 2), transient expression of Cherry‐FIT2 or GFP‐FIT2 in BY‐2 cells resulted in a marked increase in monodansylpentane (MDH)‐stained LDs in comparison with mock‐transformed control cells, and the fusion protein localized throughout concanavalin A (ConA)‐stained ER. Unlike control cells, however, the ER of cells expressing FIT2 was generally more punctate and globular in nature (Figure 3a), which is often observed for transiently expressed proteins that target to ER subdomains. Co‐expression of Cherry‐tagged FIT2 with either myc‐epitope‐tagged DGAT2 or GFP‐tagged SEIPIN1 showed clear colocalizations in specific regions of ER (Figure 3b). These associations appeared to be subdomain specific, as Cherry‐FIT2 did not colocalize with co‐expressed GFP‐tagged *Arabidopsis* SEC24, a soluble protein that localizes to subdomains of ER that serve as transport‐vesicle exit sites (Gidda *et al*., 2011; Hanton *et al*., 2007), or with a GFP‐tagged isoform of tung tree cytochrome *b*_5_ (Cb5), which is known to induce ER membrane ‘whorls’ or ‘karmallae’ when ectopically overexpressed (Hwang *et al*., 2004; Snapp *et al*., 2003) (Figure 3b). Control experiments are shown to help illustrate that each of the respective proteins is targeted to ConA‐stained ER subdomains, regardless of FIT2 co‐expression (Figure 3c). While we recognize that caution should be exercised in the interpretation of these results, the data suggest that mouse FIT2 colocalizes specifically with machinery in plant cells that is devoted to TAG biosynthesis and LD formation, and not likely indiscriminately localized to regions involved in other ER processes (e.g. vesicle export) or due to nonspecific membrane protein aggregation.

### Stable expression of FIT2 in *Arabidopsis* increases neutral lipid accumulation in both leaf and seed tissues

**Figure 4:**
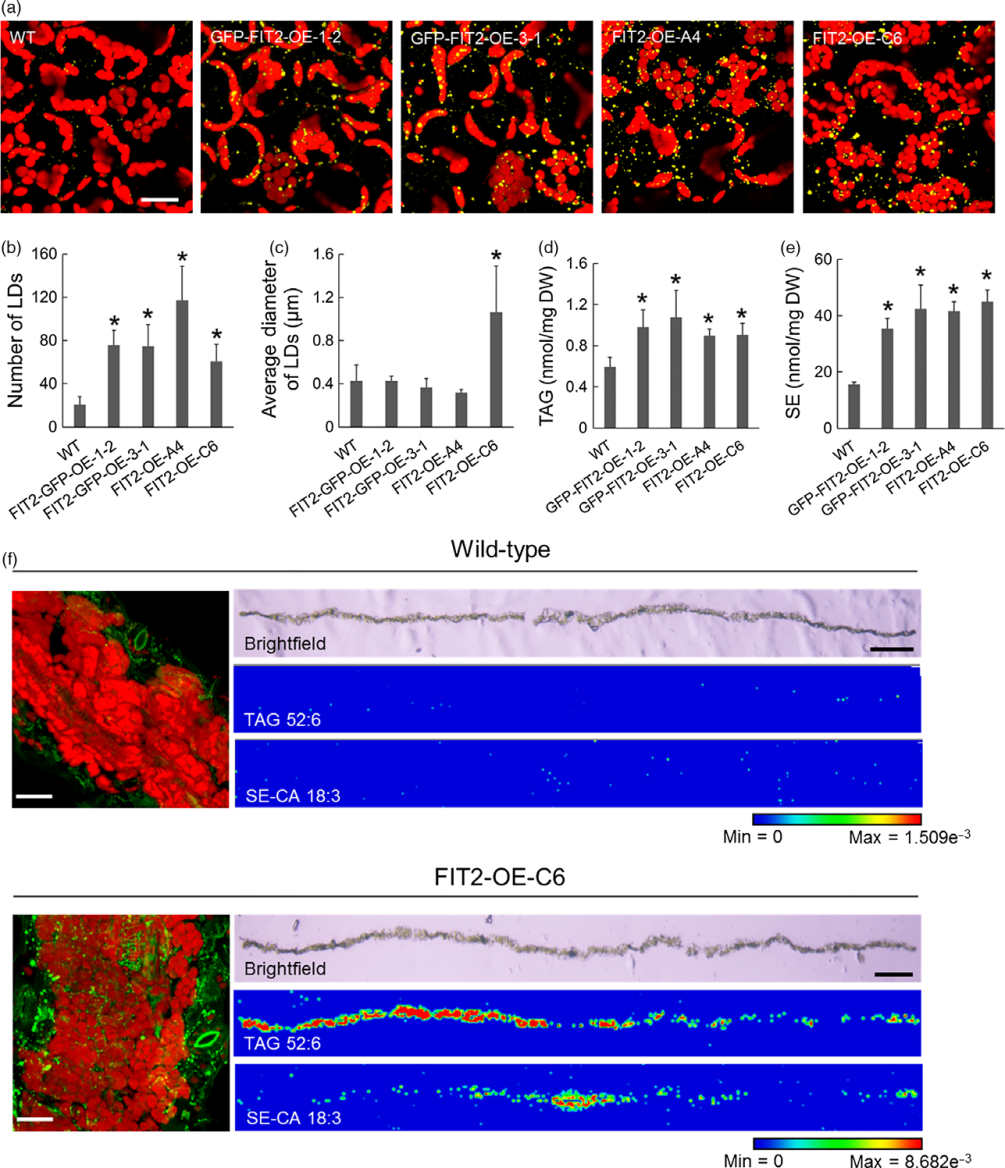
Stable expression of FIT2 in *Arabidopsis* increases LD number and neutral lipid content in leaf tissues. (a) Representative confocal images of wild‐type and FIT2 transgenic *Arabidopsis* leaves. Red colour indicates autofluorescence attributable to chloroplasts. LDs were stained with Nile red (false‐coloured yellow). Images are projections of Z‐stacks of 30 optical sections. (b) and (c) Numbers and sizes of LDs per view (image area) of *Arabidopsis* leaf mesophyll. LDs were quantified as Nile red‐stained lipid area in 2D images (as single optical slices). Averages and SDs were calculated based on nine images from at least three *Arabidopsis* plants. Asterisks indicate significant difference at *P *≤* *0.05 determined by Student's *t*‐test. (d) and (e) TAG and SE content in wild‐type and FIT2 transgenic *Arabidopsis* leaves. Averages and SDs are calculated from four biological replicates. Asterisks indicate significant difference at *P *≤* *0.05 determined by Student's *t*‐test. (f) Spatial distribution of BODIPY‐stained LDs (green), one major TAG species (TAG 52:6, m/z 889.668) and one major SE species (campesterol [CA] 18:3, m/z 699.547) in wild‐type and transgenic (i.e. FIT2‐OE‐C6) *Arabidopsi*s leaf cross sections. Left panels are representative confocal images of BODIPY‐stained LDs (green) in wild‐type and transgenic *Arabidopsis* leaf tissue. Autofluorescence of chloroplasts in confocal images are shown in red colour. Confocal images are projections of Z‐stacks. Bar = 20 μm. Right panels are bright field microscopy images of leaf sections and corresponding spatial distribution of TAG 52:6 and CA 18:3 detected by MALDI. Bar = 0.5 mm. The blue‐to‐red scale bar is used to indicate minimum and maximum ion intensity of potassiated TAG 52:6 and potassiated CA 18:3 molecules.

While mouse FIT2 appeared to localize to ER subdomains and promote LD formation in transient plant expression assays (Figures 1, 2, 3), we next determined how this protein would function in stably transformed plants. Towards this end, we generated several independent transgenic *Arabidopsis* lines ectopically expressing GFP‐tagged or nontagged mouse FIT2. Transgene expression was verified using reverse transcriptase (RT)‐PCR (Figure S3a). As shown in Figure 4, leaves of mature, 24‐day‐old transgenic plants showed significant increases in the numbers of LDs compared to nontransformed, wild‐type plants (Figure 4a and b), consistent with results obtained for transient expression in tobacco leaves and suspension cells (Figures 1, 2, 3). Further, in one line (i.e. FIT2‐OE‐C6), in addition to increased numbers of LDs, the LDs were significantly larger than those in wild‐type plants or other FIT2 lines (Figure 4c).

Figure 4 shows also that the neutral lipid content was increased in FIT2 transgenic *Arabidopsis* plant leaves (with or without GFP), with significant increases in both TAG and SE quantified by ESI‐MS/MS, in comparison with wild‐type plants (Figure 4d and e; and Figure S4; to 0.1% TAG and 3% SE on a dry weight basis). Recently, it has become possible to visualize lipid species directly in tissues by MALDI‐MS (Horn and Chapman, 2014a; Sturtevant *et al*., 2016). Hence, we confirmed *in situ* by MALDI‐MS (matrix‐assisted laser desorption/ionization MS) imaging of cryosectioned leaves that two major neutral lipid molecular species, one TAG (TAG 52:6, m/z 889.668) and one SE (Campesterol [CA] 18:3, m/z 699.547), were in fact elevated and distributed in the mesophyll tissues of FIT2 transgenic leaves, that is FIT2‐OE‐C6 (Figure 4f). Curiously, there seemed to be a somewhat heterogeneous distribution of the TAG species relative to the SE species in the leaves of this line (Figure 4g), suggesting that there is a difference in the location in leaves for the synthesis and/or accumulation of these two neutral lipid classes, but this possibility, and how FIT2 might participate in this process, needs to be examined in more detail. Nevertheless, this general distribution of elevated amounts of neutral lipids throughout the mesophyll tissues of leaves, compared to wild‐type leaves, was consistent with an increase in BODIPY 493/503‐stained LDs (Figure 4f, left) or Nile red‐stained LDs (Figure 4a) observed in mesophyll cells expressing FIT2 in *Arabidopsis* transgenic lines. We observed also that LDs were more prevalent in young and mature leaves (i.e. the 7th true leaf and the 5th true leaf from the bottom of the plant, respectively) compared with older leaves (i.e. the 3rd true leaf from the bottom of the plant) (Figure S3b), perhaps reflecting an influence of FIT2 in more metabolically active tissues where neutral lipids are likely to be synthesized. Further, transgenic leaves exhibited quantifiable increases in both TAGs and SEs, and most major molecular species of these neutral lipids increased significantly (Figure S4), indicating that FIT2 promoted a general elevation in neutral lipids that were already present in leaves, and not a specific accumulation of new or selected species. The composition of PC molecular species was not altered in a substantial way by stable, ectopic expression of FIT2 (Figure S5).

**Figure 5:**
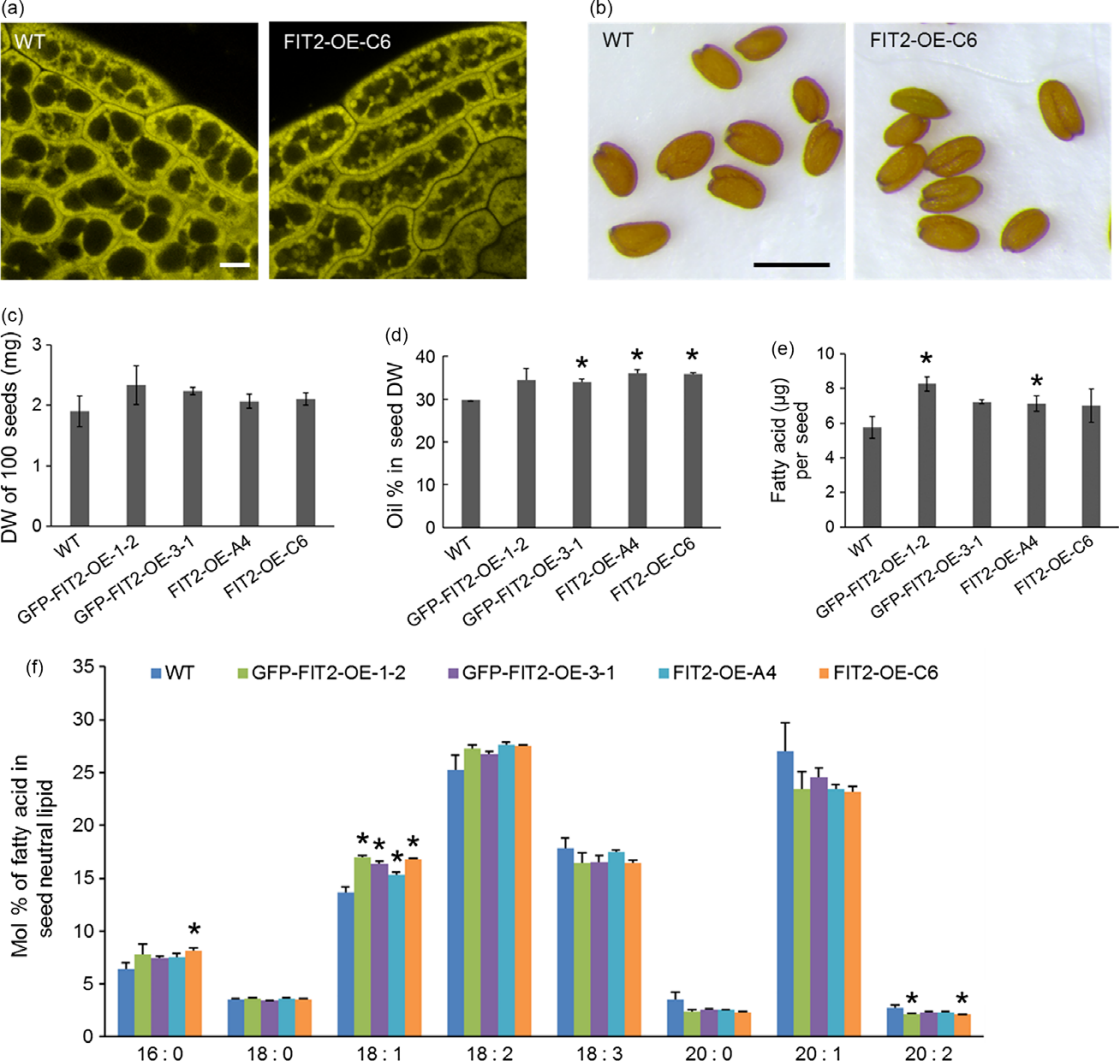
Stable FIT2 expression increases oil content in *Arabidopsis* seeds. (a) Representative confocal images of LDs in wild‐type and FIT2 transgenic (i.e. FIT2‐OE‐C6 line) *Arabidopsis* seeds. LDs were stained with Nile red (false‐coloured yellow). Bar = 5 μm. (b) Mature seeds from wild‐type and FIT2 transgenic *Arabidopsis* plants. Bar = 0.5 mm. (c) to (f) Seed size and seed oil content of wild‐type and FIT2 transgenic *Arabidopsis*. Averages and SDs were calculated from three biological replicates. Seeds were collected from *Arabidopsis* plants grown under the same conditions. Asterisks indicate significant difference at *P *≤* *0.05 determined by Student's *t*‐test. (c) Weight of 100 dry seeds. (d) Oil content in dry seeds on a weight basis measured by NMR. (e) Oil content per seed on a fatty acid basis quantified by GC‐FID. (f) Fatty acid composition in mature seeds.

While there was a significant increase in the numbers of LDs and contents of neutral lipids in vegetative tissues of *Arabidopsis* plants stably expressing mouse FIT2 (Figure 4), we also asked whether FIT2 might enhance the accumulation of TAGs in lipid‐rich seed tissues in these same lines. As shown in Figure 5a, confocal microscopy of parenchyma cells in embryo cotyledons suggested that the size and distribution of Nile red‐stained LDs was somewhat different in transgenic seeds compared to wild‐type, with larger‐sized LDs often observed within the interior of these cells (Figure 5a). Furthermore, while seed size and dry weight were not significantly altered by FIT2 expression (Figure 5b and c), there was a modest but significant increase in seed oil content in some, but not all, FIT2 transgenic lines (measured by time‐domain ^1^H‐NMR), and also in the amounts of total fatty acids per seed measured by gas chromatography‐flame ionization detection (GC‐FID) (Figure 5d and e). More specifically, when plants were grown under identical conditions, some FIT2‐expressing lines displayed an increase in seed oil content over wild‐type by as much as 13% (e.g. 34% oil versus 30% oil on a dry weight basis for FIT2‐OE‐C6 versus WT, respectively) (Figure 5d), suggesting that FIT2 expression can enhance overall oil content in seed tissues, as well as in nonseed (vegetative) tissues of plants. Furthermore, fatty acid composition of the seed oil was mostly similar between wild‐type and FIT2 lines, with only some subtle differences due to an increase in 18:1 and decrease in 20:1 acyl moieties (Figure 5f).

GFP‐tagged FIT2 was also visualized in *Arabidopsis* leaves and seeds to confirm protein expression and evaluate the localization of FIT2 relative to LDs in these tissues (Figure S6). Similar to results in tobacco leaves and suspension cells (Figures 2 and 3), in *Arabidopsis* leaves, GFP‐tagged FIT2 showed accumulations of concentrated fluorescence often in the proximity of Nile red‐stained LDs (Figure S6). In seeds, the GFP fluorescence signal was relatively weak but indicated that GFP‐FIT2 was associated with the LDs in places (Figure S6); however, the ER organization is rearranged in desiccated seed tissues (Hsieh and Huang, 2004; Wang *et al*., 2010), and perhaps the residual FIT2 persisting through seed desiccation was localized to LDs or regions of ER intimately surrounding the LDs.

## Discussion

FIT2 has been described as part of an evolutionarily conserved family of proteins that are important for the subcellular compartmentalization of neutral lipids in mammals, yeast, insects and *C. elegans* (Choudhary *et al*., 2015; Goh *et al*., 2015; Gross *et al*., 2010; Kadereit *et al*., 2008). It is curious, therefore, that this essential protein would not be present in all eukaryotes that form LDs from the ER, as no obvious homologues have been identified in plant genomes. Cytosolic LD biogenesis from the ER membrane shares conserved features at the cellular level across kingdoms, and there are likely overlapping sets of proteins that cooperate in this process, perhaps encoded by distantly related genes. Indeed, SEIPIN genes were recently identified in plant genomes, and three isoforms in *Arabidopsis* were shown to participate in the biogenesis of LDs in plant cells, similar to their function in other eukaryotes (Cai *et al*., 2015). In some cases, a shared cellular process might also be carried out by distinctly different proteins, such as the oleosins and LDAPs in plants and the perilipins in mammals, all of which bind the LD surface and stabilize LDs in the cytosol, yet share no obvious sequence similarity (Chapman *et al*., 2012; Gidda *et al*., 2016).

Here, we have asked whether a protein without any known homologues in plants might still function in a plant cell context to influence LD biogenesis. FIT2 functions, at least in part, by binding to TAG (Gross *et al*., 2011), and thus, its role in LD biogenesis is likely dependent on biophysical properties of cellular membranes that might also be present in plant cells. Notably, similar approaches were used to explore the targeting and function of oleosins, caleosins and perilipins in LD formation in yeast cells, which otherwise lack these proteins endogenously (Beaudoin and Napier, 2002; Froissard *et al*., 2009; Jacquier *et al*., 2013; Mishra and Schneiter, 2015; Rowe *et al*., 2016). Clearly, ectopic expression of mouse FIT2 in plant cells, similar to results observed in yeast and animal cells (Choudhary *et al*., 2015; Gross *et al*., 2010; Kadereit *et al*., 2008), increases production of LDs and associated TAGs (Figures 1, 2, 4 and 5). From the results presented here, it also appears that FIT2, due to its inherent ability to bind TAGs (Gross *et al*., 2011), localizes to TAG‐forming domains of the ER in plant cells where it functions to promote LD formation. SEs were also elevated in leaves expressing FIT2 (Figures 1 and 4; Figures S1 and S6), however, suggesting that FIT2 engages in and enhances a general coordination of neutral lipid synthesis and LD formation. Indeed, when co‐expressed with DGAT2 or SEIPIN1, FIT2 colocalized with both proteins (Figure 3), consistent with the premise that FIT2 associates with domains involved in both TAG synthesis and nascent LD biogenesis (Choudhary *et al*., 2015). The activity of FIT2 in plant cells suggests it might be useful as a probe for further studying the molecular nature of LD‐forming domains in plants, possibly identifying other, as yet unidentified proteins involved in LD biogenesis.

The ectopic expression of a seed‐specific transcription factor such as LEC2 in leaves has been shown by us and others to promote the accumulation of TAGs in leaf tissues (Mu *et al*., 2008; Sanjaya *et al*., 2013; Slocombe *et al*., 2009), and this is accentuated when additional proteins that promote TAG assembly, like DGAT, or proteins that bind to cytosolic LDs, like oleosins, are coproduced (Andrianov *et al*., 2010; Vanhercke *et al*., 2013, 2014; Winichayakul *et al*., 2013). Together, these engineering approaches have been described in a ‘push, pull and protect’ strategy to overproduce TAG in vegetative tissues (Vanhercke *et al*., 2014; Xu and Shanklin, 2016). In this model, seed‐specific transcription factors up‐regulate the fatty acid biosynthetic machinery (Grimberg *et al*., 2015), effectively ‘pushing’ reduced photosynthetic carbon into lipid, up‐regulation of DGAT promotes the synthesis of TAGs at the ER, ‘pulling’ reduced carbon into TAG, and oleosins ‘protect’ the sequestered TAG from the active metabolic pool and prevent the hydrolysis of TAGs and turnover of LDs. In fact, remarkable amounts of TAG have been accumulated in leaves of transgenic tobacco plants—up to more than 15% dry weight of leaves—using a combination of these engineering processes (Vanhercke *et al*., 2014). The increase in the energy density of leaves (i.e. increased lipid content relative to carbohydrate) will likely find multiple applications in agriculture and bioenergy, such as higher calorie and nutritionally balanced feeds, or as a source of oleochemicals and biodiesel feedstock (Horn and Benning, 2016). Indeed, it has been suggested that an increase in vegetative biomass of lipid up to 10% dry weight would result in a 30% increase in usable liquid fuels, through a combination of both biodiesel and lignocellulosic‐derived fuels (Ohlrogge and Chapman, 2011). The production of large amounts of lipids in vegetative tissues makes possible the separation of calories in edible oilseeds needed for human nutrition from a potential bioenergy feedstock derived from vegetative biomass, preventing renewable sources of energy from competing with food production. What remains to be explored is how proteins involved in packaging TAG into LDs, such as SEIPIN, FIT2 (or its yet‐to‐be identified functionally equivalent plant orthologue), might complement ongoing approaches to enhance the energy content of plant vegetative tissues. Certainly, it seems that co‐expression of LEC2 with variants of FIT2 in plant leaves can result in marked increase of compartmentalized LDs and neutral lipid content above that seen with either LEC2 or FIT2 alone (Figure 1), and so perhaps some component of LD ‘release’ would add significantly to the ‘push–pull–protect’ theory of increasing lipid accumulation in plant vegetative tissues.

## Experimental procedures

### Plant material, growth conditions and transformation

*A. thaliana* (Columbia‐0) plants were grown in growth chambers on either one‐half‐strength MS‐media‐containing plates (Murashige and Skoog, 1962) or in soil at 21 °C under a 16/8‐h light/dark cycle with 50 μE/m^2^/s light intensity. *Nicotiana benthamiana* plants were grown in soil at 28 °C under a 14/10‐h light/dark cycle. Plants used in the same experiments were grown concurrently, in the same conditions. Plasmids were transformed into *Agrobacterium tumefaciens* (GV3101) by electroporation (Bio‐Rad), and stable transformation of *Arabidopsis* plants was carried out using the floral dip method (Clough and Bent, 1998). Gene expression was evaluated in transgenic *Arabidopsis* seedlings (progeny) using RT‐PCR, and independent lines were selected for further study. Infiltration of *N. benthamiana* leaves was carried out as described previously (Cai *et al*., 2015; Petrie *et al*., 2010). Tomato Bushy Stunt Virus (TBSV) *P19* was included in all infiltrations to suppress transgene silencing and intensify transgene expression (Petrie *et al*., 2010); *Arabidopsis LEC2* was included in some co‐infiltrations to promote seed‐like lipid synthesis in leaves (Cai *et al*., 2015; Gidda *et al*., 2016; Santos Mendoza *et al*., 2005). *Nicotiana tabacum* Bright Yellow (BY)‐2 suspension‐cultured cells were maintained and prepared for (co)transformation via biolistic particle bombardment using a Bio‐Rad PDS system 1000/HE, as described previously (Lingard *et al*., 2008).

### Plasmid construction

The cDNA of *M. musculus FIT2* (EMBL Accession No. BAE37420.1) was kindly donated by David Silver (Duke—National University of Singapore). The *FIT2* ORF was subcloned into the plant binary expression plasmids pMDC32 and pMDC43 [Curtis and Grossniklaus, 2003; ] using restriction enzymes *Asc*I and *Pac*I, yielding pMDC32/FIT2 and pMDC43/GFP‐FIT2, respectively. Both pMDC32 and pMDC43, as well as all other plant expression plasmids used in this study, contain the CaMV 35S constitutive promoter (Benfey *et al*., 1989). Mutated versions of *FIT2* (i.e. FIT2‐N^[80]^A and FIT2‐FLL^[157‐159]^AAA) were generated using PCR‐fusion cloning procedures (Atanassov *et al*., 2009) and then subcloned into pMDC32 and pMDC43. pRTL2/Cherry‐FIT2 was constructed by subcloning the *FIT2* ORF into the transient expression plasmid pRTL2/Cherry‐MCS, which contains the Cherry fluorescent protein coding sequence (Gidda *et al*., 2011). Plant binary expression plasmids containing *Arabidopsis LEC2* (pORE04‐*LEC2*) and *Tomato Bushy Stunt Virus P19* (pORE04‐*P19)* were provided by Qing Liu (CSIRO) (Petrie *et al*., 2010). Other plant expression plasmids, including pMDC32/CFP‐HDEL, pMDC43/GFP‐SEIPIN1, pRTL2/GFP‐SEC24, pRTL2/Myc‐DGAT2 and pRTL2/GFP‐Cb5, have been described elsewhere (Cai *et al*., 2015; Gidda *et al*., 2011; Hwang *et al*., 2004; Shockey *et al*., 2006).

### Rt‐pcr

RNA was extracted from 4‐week‐old *Arabidopsis* plants using the RNeasy Plant Mini Kit (Qiagen, Germantown, MD, USA), and DNA contamination was eliminated using DNase (Promega, Madison, WI, USA). Approximately 100 ng of total RNA was used for RT‐PCRs, which were performed with TaKaRa One Step RT‐PCR Kit following the manufacturer's instructions. The PCR program was as follows: 42 °C for 15 min, 95 °C for 5 min, 35 amplification cycles (94 °C for 30 s, 55 °C for 30 s, 72 °C 30–60 s), and 72 °C for 7 min. Primers used to amplify *Arabidopsis EF1*α were those used elsewhere (Cai *et al*., 2015). Primers used to amplify mouse *FIT2* were FIT2RTF 5′‐ATGGAGCACCTGGAGCGC‐3′ and FIT2RTR 5′‐ TCATTTCTTGTAAGTATCTCGCTTCAAAG‐3′.

### Lipid analysis

For neutral lipid content and compositional analysis, ~45 mg dry weight of *N. benthamiana* leaves, 15 mg of *Arabidopsis* leaves (dry weight) or 10 mg of *Arabidopsis* seeds were used in each replicate. Sample tissues were homogenized with glass beads in a bead beater (BioSpec Mini‐Beadbeater‐16) with 70 °C isopropanol. TAG (tri‐17:0) standard (Avanti) was added to *Arabidopsis* seed samples, while TAG (tri‐15:0) standard and cholesterol ester (C13:0) standard (Nu‐Chek Prep) were added to leaf samples. Total lipid was extracted as described by Cai *et al*. (2015). The neutral lipid fraction and polar lipid fraction were separated from total leaf lipid extract by solid‐phase extraction using 6‐mL silica columns (Sigma‐Aldrich, St. Louis, MO, USA). Hexane and diethyl ether (4:1) were used to elute neutral lipids, and chloroform and methanol (1:2) were used to elute polar lipids. PC (di‐14:0) (Avanti) standard was added to the polar lipid fraction of *Arabidopsis* leaf samples. Lipid content in *Arabidopsis* (TAG, SE and PC) and *N. benthamiana* (TAG and SE) leaves was analysed by electrospray ionization mass spectrometry (ESI‐MS) on an API3000 triple quadrupole mass spectrometer (Applied Biosystems/Sciex, Framingham, MA), and data were processed using Metabolite Imager (v.1.0) to quantify the TAG and SE content (Horn and Chapman, 2014b; Li *et al*., 2014) and using LipidomeDB Data Calculation Environment to quantify the PC content (Welti *et al*., 2002; Zhou *et al*., 2011). Lipid extract from *Arabidopsis* seeds was transesterified with methanolic HCl, and the fatty acid methyl esters were analysed using gas chromatography–flame ionization detection (GC‐FID, Hewlett Packard, HP 5890 II plus GC). Conditions for both ESI‐MS and GC‐FID were as described in James *et al*. (2010). Total oil content of *Arabidopsis* seeds was quantified using time‐domain, pulse‐field ^1^H‐NMR with a Bruker Minispec mq20 (Bruker optics), as described by Chapman *et al*. (2008), except calibrated for small volumes of *Arabidopsis* seeds.

Spatial distributions of TAG and SE in *Arabidopsis* leaves were determined *in situ* by matrix‐assisted laser desorption/ionization mass spectrometry imaging (MALDI‐MSI). *Arabidopsis* leaves were embedded in porcine gelatin and then frozen to −80 °C, before equilibration to −20 °C. Tissues were cut into 40‐μm‐thick cross sections on a cryostat (Leica CM1950) at −18 °C and lyophilized overnight before application of matrix (2,5‐dihydroxybenzoic acid) by sublimation. A MALDI Orbitrap hybrid mass spectrometer (MALDI LTQ Orbitrap‐XL; Thermo Fisher Scientific, Waltham, MA, USA) was used to scan the tissue sections at 40 micron steps, and the MS data were used to reconstruct false‐colour MALDI‐MSI images based on ion counts of selected lipid species (Horn and Chapman, 2014b). Lipid species were identified by exact mass comparisons with the LIPID MAPS database (http://www.lipidmaps.org/). Detailed procedures for data acquisition and analyses were described previously (Horn *et al*., 2012).

### Microscopy

Confocal images of *Arabidopsis* leaves and seeds and *N. benthamiana* leaves were obtained using a Zeiss LSM 710 confocal laser‐scanning microscope (CLSM). Images of *N. tabacum* (BY‐2) cells were obtained using either a Leica DM RBE microscope equipped with a Leica TCS SP2 scanning head or a Leica SP5 CLSM system equipped with a Radius 405‐nm laser. *Arabidopsis* leaves and seeds, as well as tobacco leaves, were processed for CLSM imaging, including staining of LDs with the neutral lipid stains BODIPY 493/503 (Invitrogen) or Nile red (Sigma‐Aldrich), as described previously (Cai *et al*., 2015; Gidda *et al*., 2016; Park *et al*., 2013). The fifth leaf from the bottom of 24‐day‐old *Arabidopsis* plants was collected for analysis. *Arabidopsis* leaves used in comparison with young, mature and older mature leaves were the seventh, fifth and third true leaf from the bottom of the plant, respectively. Three fully expanded leaves from the top of the *N. benthamiana* plants (4‐week‐old) were used in the transient expression experiments. BY‐2 cells were processed for CSLM as described previously (Gidda *et al*., 2011, 2016; Lingard *et al*., 2008). Briefly, cells ~4–6 h following biolistic bombardment were fixed in paraformaldehyde (Electron Microscopy Sciences), permeabilized and then incubated with the ER stain fluor‐conjugated concanavalin A (ConA) (Sigma‐Aldrich) and/or the neutral lipid stain MDH (Abgent). Cherry was excited with a 543‐nm laser, and emission signals were collected from 590 to 640 nm. BODIPY 493/503, GFP and Nile red, as well as chlorophyll, were excited with a 488‐nm laser, and emission signals were acquired from 500 to 540 nm (BODIPY and GFP), 560 to 620 nm (Nile red) and 640 to 720 nm (chlorophyll), respectively. CFP and MDH were excited with a 405‐nm laser, and the emission signal was collected from 450 to 490 nm and 420 to 480 nm, respectively. All fluorophore emissions were collected sequentially in double‐ or triple‐labelling experiments; single‐labelling experiments showed no detectable crossover at the settings used for image acquisition. Depending on the plant material, images were acquired as a Z‐series (leaves of *Arabidopsis* and *N. benthamiana*) or single optical sections (*Arabidopsis* seeds and BY‐2 cells). All fluorescence images of cells shown are representative of at least two separate experiments, including at least three independently infiltrated leaves from three *N. benthamiana* plants or 20 transiently transformed BY‐2 cells. The numbers and sizes of LDs in *Arabidopsis* and *N. benthamiana* leaves were quantified as BODIPY‐ or Nile red‐stained lipid area according to Cai *et al*. (2015) using the Analyze Particles function at ImageJ (version 1.43). All the significance assessments in this study were performed using Student's *t*‐test.

## Supporting information


**Figure S1** Composition of TAG (a) and SE (b) in *Nicotiana benthamiana* leaves expressing FIT2 or FIT2‐FLL^[157‐159]^AAA in the presence or absence of LEC2.
**Figure S2** Localization of GFP‐FIT2 to the ER in *N. tabacum* suspension‐cultured BY‐2 cells.
**Figure S3** Expression of *FIT2* in transgenic *Arabidopsis* leaves and influence of ectopically‐expressed FIT2 on LDs in different‐aged leaves of *Arabidopsis* plants.
**Figure S4** Composition of TAG (a) and SE (b) in wild‐type and FIT2 transgenic (i.e., FIT2‐OE‐C6) *Arabidopsis* leaves.
**Figure S5** Composition of PC molecular species in wild‐type and FIT2 transgenic *Arabidopsis* leaves.
**Figure S6** Visualization of GFP‐FIT2 and LDs in leaf and seed tissues of stable transgenic *Arabidopsis*.
