# Supplementary material for: Mouse fat storage‐inducing transmembrane protein 2 (FIT2) promotes lipid droplet accumulation in plants
Source: Plant Biotechnol J. 2017 Jan 18;15(7):824–36. doi: 10.1111/pbi.12678 (PMC5466434; doi:10.1111/pbi.12678)
Supplement: Supplementary file 1 — Figure S1 Composition of TAG (a) and SE (b) in Nicotiana benthamiana leaves expressing FIT2 or FIT2‐FLL[157‐159]AAA in the presence or absence of LEC2. Figure S2 Localization of GFP‐FIT2 to the ER in N. tabacum suspension‐cultured BY‐2 cells. Figure S3 Expression of FIT2 in transgenic Arabidopsis leaves and influence of ectopically‐expressed FIT2 on LDs in different‐aged leaves of Arabidopsis plants. Figure S4 Composition of TAG (a) and SE (b) in wild‐type and FIT2 transgenic (i.e., FIT2‐OE‐C6) Arabidopsis leaves. Figure S5 Composition of PC molecular species in wild‐type and FIT2 transgenic Arabidopsis leaves. Figure S6 Visualization of GFP‐FIT2 and LDs in leaf and seed tissues of stable transgenic Arabidopsis. [file PBI-15-824-s001.pptx]

## Slide 1
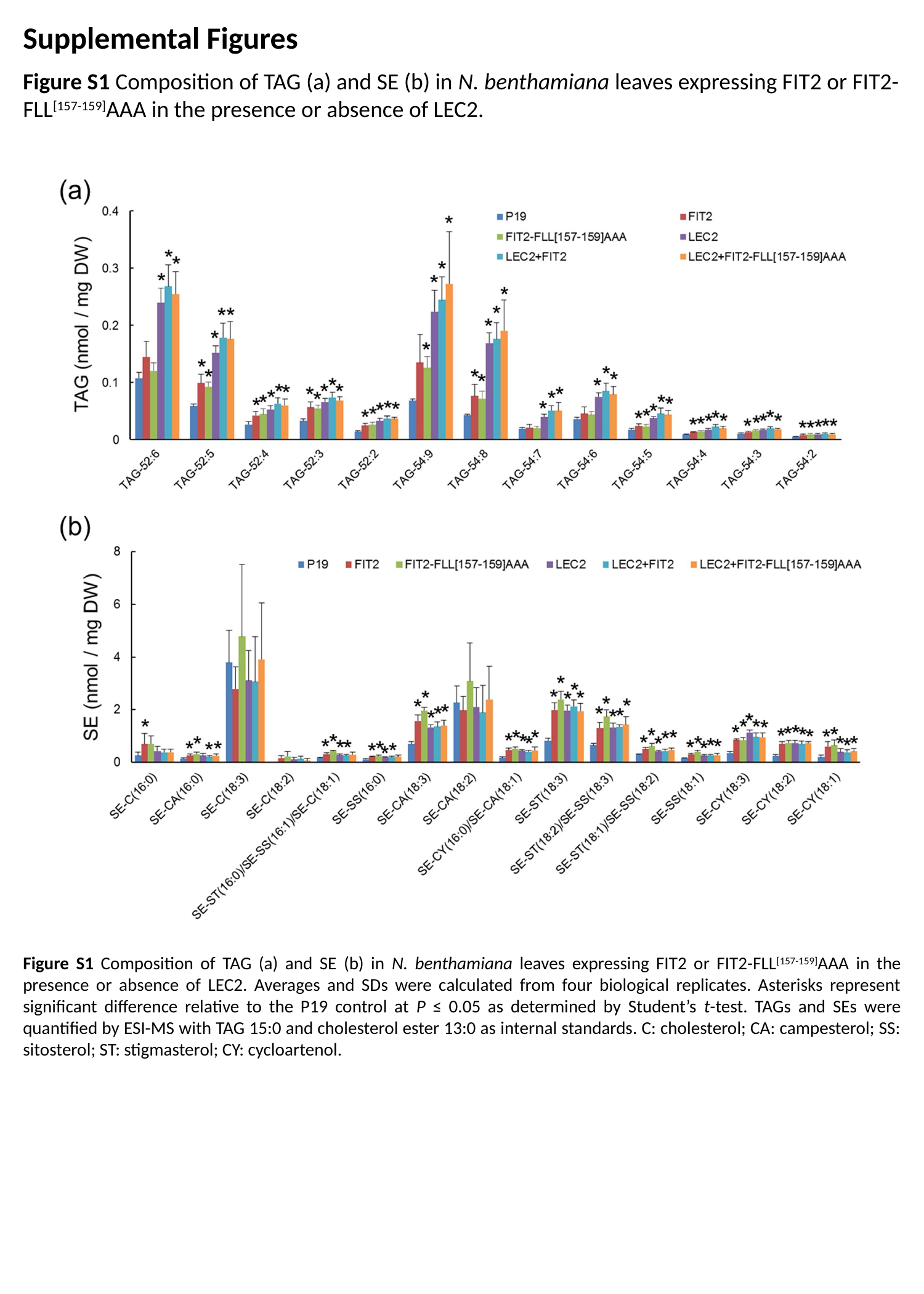

Supplemental Figures
Figure S1 Composition of TAG (a) and SE (b) in N. benthamiana leaves expressing FIT2 or FIT2-FLL[157-159]AAA in the presence or absence of LEC2.
Figure S1 Composition of TAG (a) and SE (b) in N. benthamiana leaves expressing FIT2 or FIT2-FLL[157-159]AAA in the presence or absence of LEC2. Averages and SDs were calculated from four biological replicates. Asterisks represent significant difference relative to the P19 control at P ≤ 0.05 as determined by Student’s t-test. TAGs and SEs were quantified by ESI-MS with TAG 15:0 and cholesterol ester 13:0 as internal standards. C: cholesterol; CA: campesterol; SS: sitosterol; ST: stigmasterol; CY: cycloartenol.

## Slide 2
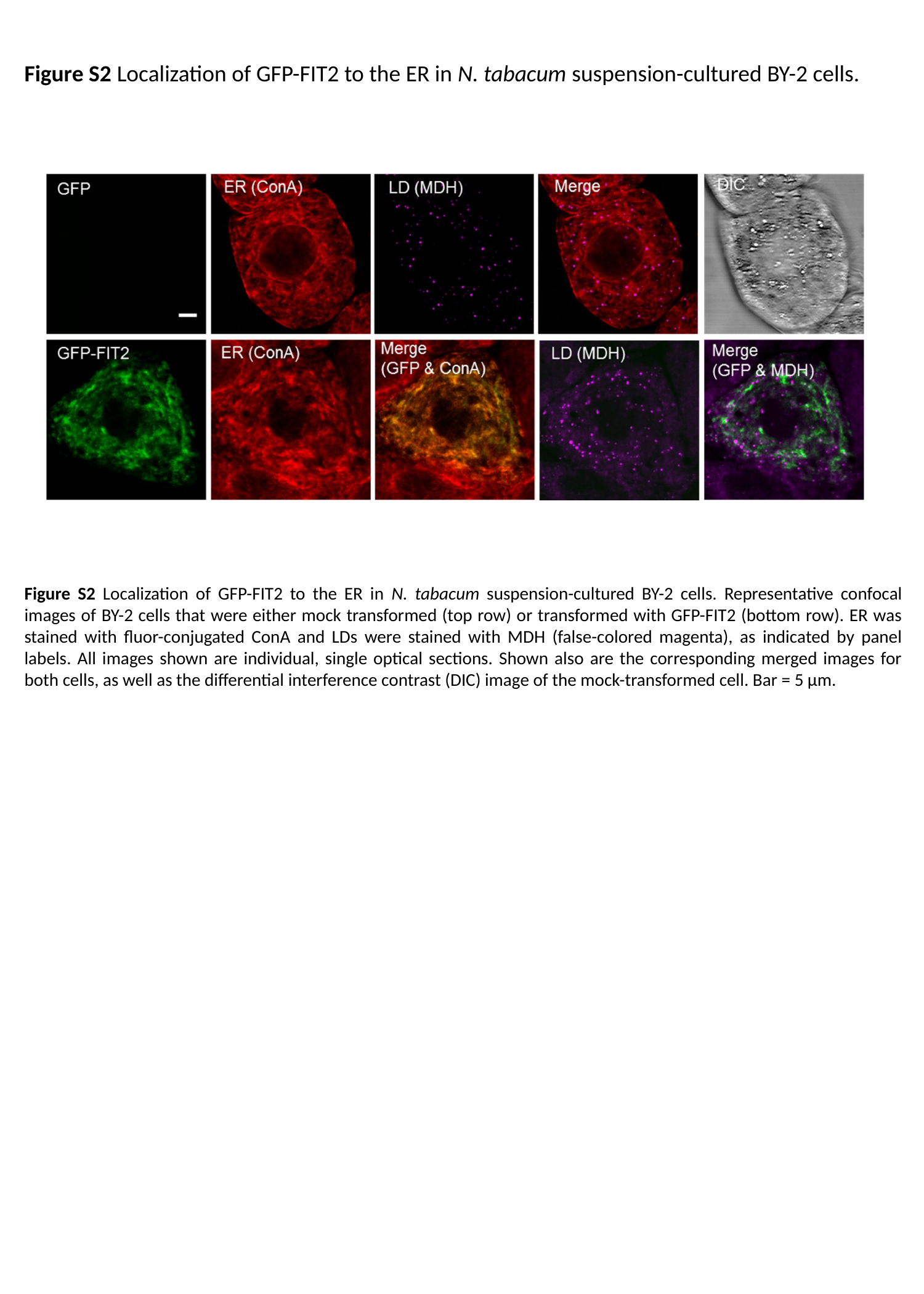

Figure S2 Localization of GFP-FIT2 to the ER in N. tabacum suspension-cultured BY-2 cells.
Figure S2 Localization of GFP-FIT2 to the ER in N. tabacum suspension-cultured BY-2 cells. Representative confocal images of BY-2 cells that were either mock transformed (top row) or transformed with GFP-FIT2 (bottom row). ER was stained with fluor-conjugated ConA and LDs were stained with MDH (false-colored magenta), as indicated by panel labels. All images shown are individual, single optical sections. Shown also are the corresponding merged images for both cells, as well as the differential interference contrast (DIC) image of the mock-transformed cell. Bar = 5 µm.

## Slide 3
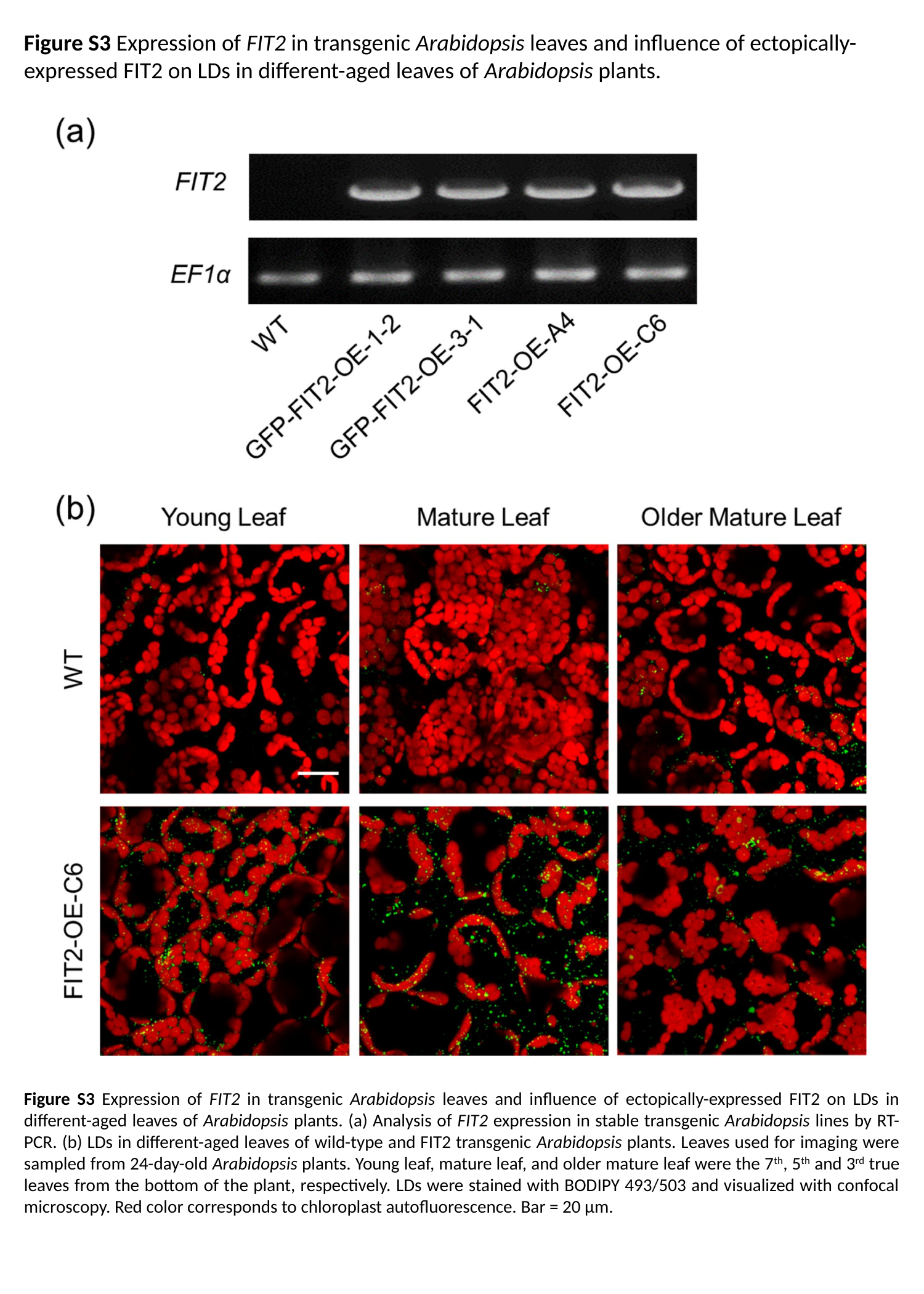

Figure S3 Expression of FIT2 in transgenic Arabidopsis leaves and influence of ectopically-expressed FIT2 on LDs in different-aged leaves of Arabidopsis plants.
Figure S3 Expression of FIT2 in transgenic Arabidopsis leaves and influence of ectopically-expressed FIT2 on LDs in different-aged leaves of Arabidopsis plants. (a) Analysis of FIT2 expression in stable transgenic Arabidopsis lines by RT-PCR. (b) LDs in different-aged leaves of wild-type and FIT2 transgenic Arabidopsis plants. Leaves used for imaging were sampled from 24-day-old Arabidopsis plants. Young leaf, mature leaf, and older mature leaf were the 7th, 5th and 3rd true leaves from the bottom of the plant, respectively. LDs were stained with BODIPY 493/503 and visualized with confocal microscopy. Red color corresponds to chloroplast autofluorescence. Bar = 20 µm.

## Slide 4
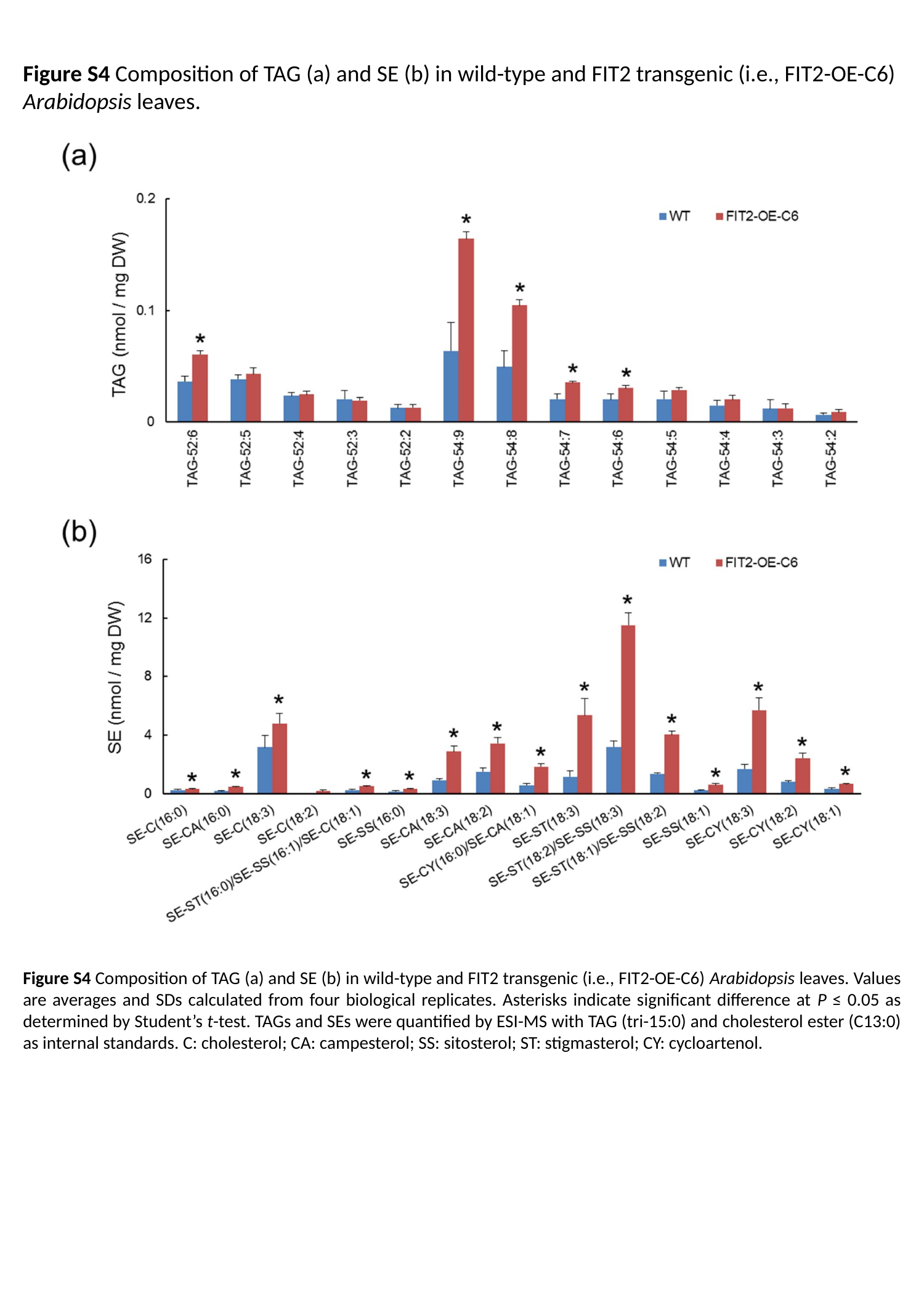

Figure S4 Composition of TAG (a) and SE (b) in wild-type and FIT2 transgenic (i.e., FIT2-OE-C6) Arabidopsis leaves.
Figure S4 Composition of TAG (a) and SE (b) in wild-type and FIT2 transgenic (i.e., FIT2-OE-C6) Arabidopsis leaves. Values are averages and SDs calculated from four biological replicates. Asterisks indicate significant difference at P ≤ 0.05 as determined by Student’s t-test. TAGs and SEs were quantified by ESI-MS with TAG (tri-15:0) and cholesterol ester (C13:0) as internal standards. C: cholesterol; CA: campesterol; SS: sitosterol; ST: stigmasterol; CY: cycloartenol.

## Slide 5
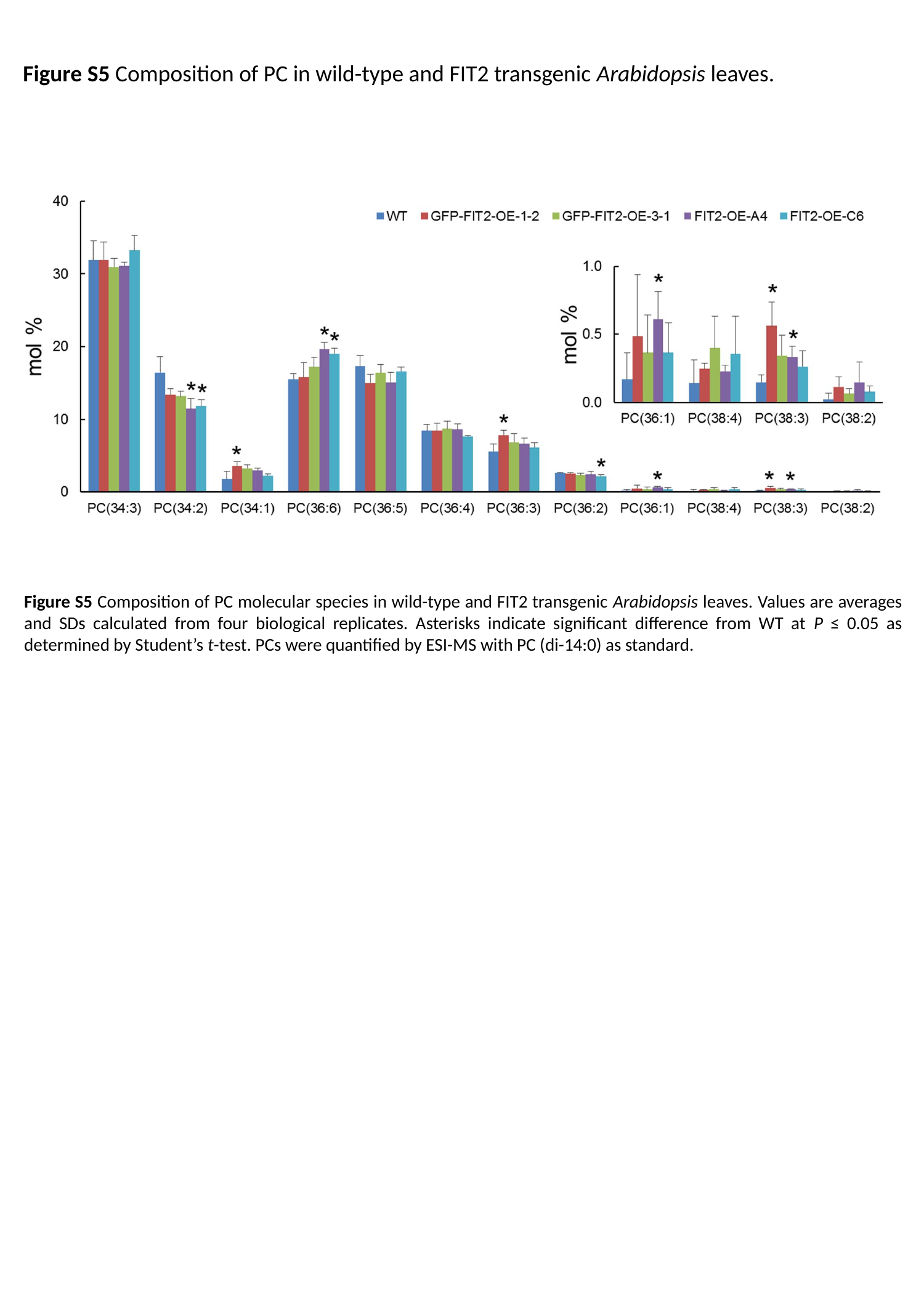

Figure S5 Composition of PC in wild-type and FIT2 transgenic Arabidopsis leaves.
Figure S5 Composition of PC molecular species in wild-type and FIT2 transgenic Arabidopsis leaves. Values are averages and SDs calculated from four biological replicates. Asterisks indicate significant difference from WT at P ≤ 0.05 as determined by Student’s t-test. PCs were quantified by ESI-MS with PC (di-14:0) as standard.

## Slide 6
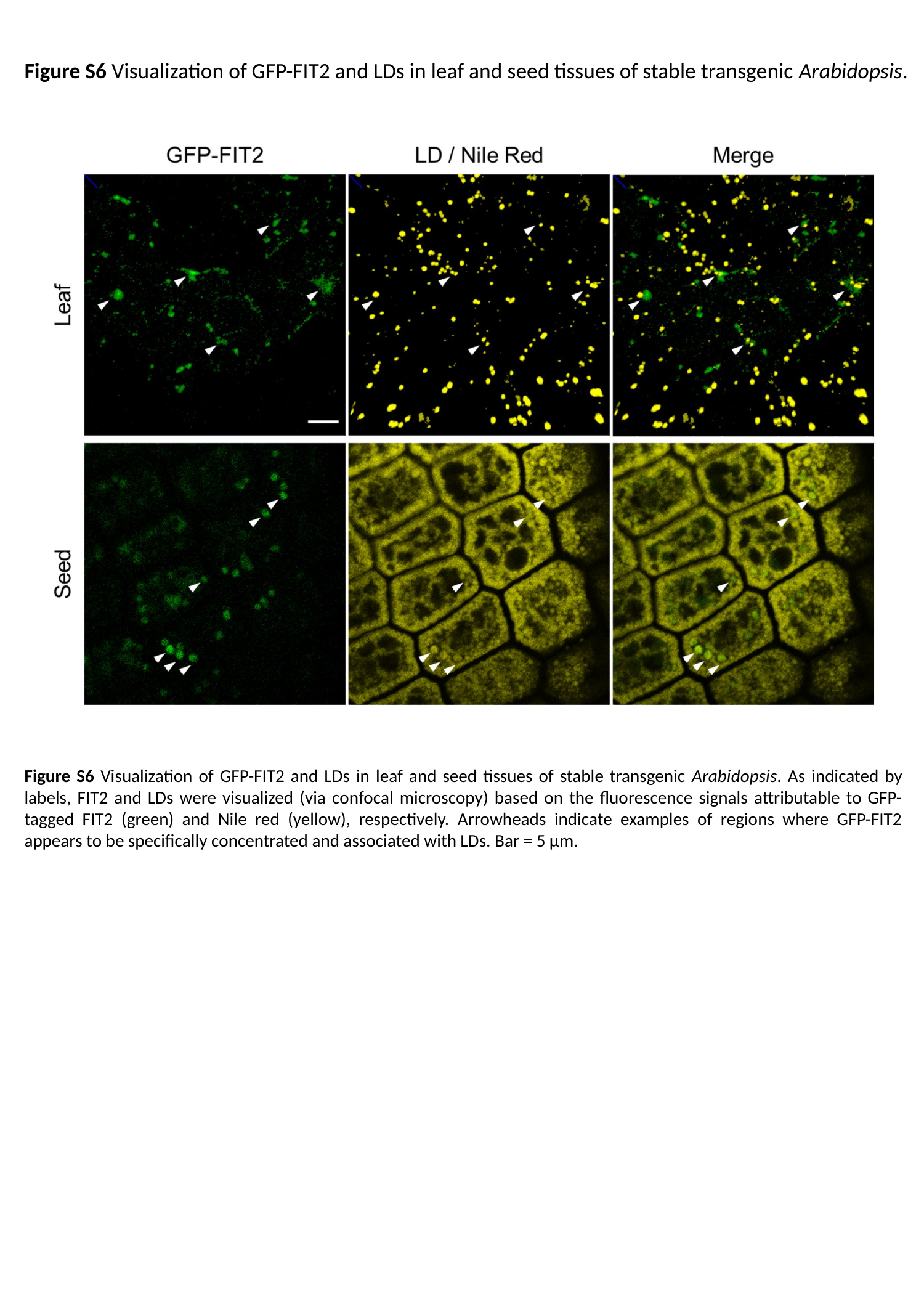

Figure S6 Visualization of GFP-FIT2 and LDs in leaf and seed tissues of stable transgenic Arabidopsis.
Figure S6 Visualization of GFP-FIT2 and LDs in leaf and seed tissues of stable transgenic Arabidopsis. As indicated by labels, FIT2 and LDs were visualized (via confocal microscopy) based on the fluorescence signals attributable to GFP-tagged FIT2 (green) and Nile red (yellow), respectively. Arrowheads indicate examples of regions where GFP-FIT2 appears to be specifically concentrated and associated with LDs. Bar = 5 µm.
